# Combining Electrodermal Activity With the Peak-Pain Time to Quantify Three Temporal Regions of Pain Experience

**DOI:** 10.3389/fpain.2022.764128

**Published:** 2022-03-23

**Authors:** Viprali Bhatkar, Rosalind Picard, Camilla Staahl

**Affiliations:** ^1^Digital Health Independent Consultant, Arlington, MA, United States; ^2^MIT Media Lab, Cambridge, MA, United States; ^3^Novo Nordisk A/S, R&D Business Development, Copenhagen, Denmark

**Keywords:** sympathetic nervous system, electrodermal, EDA, SCL, VAS, cold pressor, capsaicin

## Abstract

**Background:**

Self-reported pain levels, while easily measured, are often not reliable for quantifying pain. More objective methods are needed that supplement self-report without adding undue burden or cost to a study. Methods that integrate multiple measures, such as combining self-report with physiology in a structured and specific-to-pain protocol may improve measures.

**Method:**

We propose and study a novel measure that combines the *timing of the peak pain* measured by an electronic visual-analog-scale (eVAS) with continuously-measured changes in electrodermal activity (EDA), a physiological measure quantifying sympathetic nervous system activity that is easily recorded with a skin-surface sensor. The new pain measure isolates and specifically quantifies three temporal regions of dynamic pain experience: I. Anticipation preceding the onset of a pain stimulus, II. Response rising to the level of peak pain, and III. Recovery from the peak pain level. We evaluate the measure across two pain models (cold pressor, capsaicin), and four types of treatments (none, A=pregabalin, B=oxycodone, C=placebo). Each of 24 patients made four visits within 8 weeks, for 96 visits total: A training visit (TV), followed by three visits double-blind presenting A, B, or C (randomized order). Within each visit, a participant experienced the cold pressor, followed by an hour of rest during which one of the four treatments was provided, followed by a repeat of the cold pressor, followed by capsaicin.

**Results:**

The novel method successfully discriminates the pain reduction effects of the four treatments across both pain models, confirming maximal pain for no-treatment, mild pain reduction for placebo, and the most pain reduction with analgesics. The new measure maintains significant discrimination across the test conditions both within a single-day's visit (for relative pain relief within a visit) and across repeated visits spanning weeks, reducing different-day-physiology affects, and providing better discriminability than using self-reported eVAS.

**Conclusion:**

The new method combines the subjectively-identified time of peak pain with capturing continuous physiological data to quantify the sympathetic nervous system response during a dynamic pain experience. The method accurately discriminates, for both pain models, the reduction of pain with clinically effective analgesics.

## Introduction

Pain involves a subjective experience influenced by factors such as fear, emotion, anxiety, cognitions, autonomic responses and malaise (1). Self-reported pain intensity does not correlate well with the severity of the pathological condition (2). Thus, quantification of analgesic effects in clinical trials, even with established analgesics, is frequently inconclusive (3).

Today's standard for pain measurement, the visual-analog scale (VAS) or electronic VAS (eVAS), allows participants to self-report their subjective experience of pain either statically–by reporting a single number, or dynamically–by turning a dial or moving a slider, usually along a scale from 1 to 100. While such scales have become the gold standard, being quick and easy to use, they have long been recognized to have problems with accuracy and reliability, with many factors beyond pain that influence the scores people give (1, 4). While many efforts are made to optimize self-report measures, e.g., customizing how it is presented for a particular population such as older adults (5), a holy grail of pain measurement is to obtain a more objective measure that sensitively reflects changes in pain experience and is easy to use. It also needs to work reliably and repeatably, across different participants experiencing different levels of pain in a lab study, and also on different days and visits, with repositioned equipment making valid measures, across different types of pain and analgesic use.

Recent surveys reviewed a growing number of automated methods to quantify pain objectively using facial expressions, vocalizations, physiology, brain-activity sensing, and more, and indicated the need for personalization of measures (6), as well as many wearable sensing approaches that can help quantify pain more objectively (7). While all of these measures show promise, each fully-objective method has its limitations, typically ignoring user-dependent subjective information, and focusing only on the objective data for one type of pain model and only during one day's visit or assessment period. The same emotions in the same person can exhibit patterns of physiology that change from day to day (8), so it is important to make sure that any pain-sensing method can account for this day-based variation.

Methods to elicit pain in a controlled manner have been refined via a large number of human pain models (9). In this work we use two well-established methods to induce pain: (1) the cold pressor, placing a limb into icy-cold water and holding it there, known for deep intense pain activating the descending pain system and its sensitivity to opioids (10), and (2) intradermal injection of capsaicin, which generates stable, long-lasting, and reproducible primary and secondary hyperalgesia lasting 2 to 3 h (11–13).

While many attempts have been made to develop an objective measure of pain, we focus in this work on a new measure that can be used easily and efficiently deployed in a variety of environments including the emergency room, post-operative recovery space, etc. This requirement rules out EEG, MRI, MEG, and fNIRS, despite that there has been exciting progress with these brain-based methods, e.g., (14, 15). We choose a measure that can be assessed as easily as vitals are assessed today with a readily applied wearable sensor, and measure the sympathetic nervous system response using a new characterization of electrodermal activity (EDA), which can be obtained quickly and easily by placing a sensor on the surface of the wrist or lower leg. The sensor can optionally be worn for continuous monitoring 24/7. Unlike the heart, which receives both sympathetic and parasympathetic nervous system innervation, the skin receives only sympathetic innervation (16). EDA thus provides a sensitive measure of sympathetic nervous system activity that can be captured effortlessly, and that changes continuously during a pain experience.

Sometimes EDA is considered non-specific, because it can be influenced by changing humidity and sweating, hydration, and strong emotions. Our method addresses the specificity problem by synchronizing the quantification of the EDA temporally to two precisely defined moments: (a) the moment of onset when applying a painful stimulus, and (b) the moment, identified subjectively, of “peak pain” experience. By using relative values in the regions anchored by these time points, EDA-based measures are likely to be highly specific to pain because they occur during an elicited experience of pain, acknowledged by a self-reported peak pain. Also, changes due to humidity, sweating, hydration, and emotions-unrelated-to-pain are minimal within the time-frame measured. We evaluate the proposed measure across people, across different days, across two pain models, and across three treatments, showing it addresses these traditional concerns.

While objective physiological data often have the strong and helpful property of being able to be continuously measured, sometimes they are limited because they change only at a *subset* of the moments of interest during a pain experience; for example, facial expressions might be most likely to occur at the onset of a cold pressor task, but the expression might fade or disappear completely, even as pain continues to increase, an observation identified decades ago (17). We seek in this work to develop a measure that continuously represents the trajectory of pain's anticipation, response, and decay.

While the subjectively reported levels provided on a VAS or eVAS can vary because of many factors unrelated to severity of pain, it is still routinely used. In our work, we use it in a way that extracts the timing of its peak, but then we discard the actual eVAS values. More specifically, when self-reporting pain, the exact value selected is highly subjective: it might be low simply because the participant wants to appear stoic. However, when a dial is turned continuously after a painful stimulus, it usually will increase up to a point, before it falls. Thus, each participant shows a moment of peak pain—the highest value relative to their other values. In our work, we find that the time to arrive at this peak is stable across pain sessions, even on different days with different pain treatments. The temporal position of the peak eVAS value is used to delineate two regions: The region rising up to this peak, and the region recovering from this peak. Our new measure then quantifies the EDA in these regions.

We also choose to include in our measure one more region: the assessment of the physiology during a period of anticipation immediately before the pain onset. This choice was inspired by hearing pediatric nurses discuss how some children flinch as if in pain or utter “ouch” *before* the needle touches them and by work showing that pre-pain anxiety can predict self-reported pain (18). Quantifying this pre-pain anxiety is not typically done in pain research, but we think it is important for better understanding patient pain experiences and we recommend its measurement, at least as a contextualizing factor before the actual pain stimulus occurs.

To summarize, the proposed new method precisely characterizes and quantifies physiology over three temporal regions:

I (Anticipation): From the announcement of an imminent painful stimulus to the pain stimulus onset
II (Response) From the stimulus onset to the moment of subjectively reported peak pain
III (Recovery) From the peak pain moment to recovery from pain, or for a fixed time after the peak

These three quantities characterize our three-region pain measure.

It is well-established that pain should be highest during a painful-stimulus condition when no treatment is provided, reduced slightly under a placebo treatment, and reduced the most by effective analgesics. On placebo effectiveness, see for example Colloca and Barsky (19) and also demonstrations that higher-priced placebos work better than lower-priced ones (20). Using this knowledge, we test the novel three-region measure in a rigorous study with a 3-armed, placebo-controlled, randomized crossover trial design including 24 healthy adults. We systematically compare each measure before and after the effects of placebo, oxycodone and pregabalin. We also examine temporal situations known to affect pain measures, including the heightened anxiety expected during a “first visit,” which can be expected to translate into a report of higher pain on the first time than when the identical procedure is repeated later. Finally, we show that the new measure outperforms the eVAS in all of these tests, demonstrating excellent pain discriminability.

## Methods

The methods used in this study are designed to evaluate a new measure of pain in the context of a clinical trial setting. We use treatments of previously established efficacy against pain (pregabalin, oxycodone) in a design of a randomized control trial. The trial applies a double-blind placebo-controlled multi-treatment, multi-day design. Outcomes were compared for all treatments both within and across participants, across days and weeks, and across different placements of the sensors, in order to comprehensively evaluate if the new pain measure is robust to all of these important variations. All study procedures were pre-approved by an ethics review board and the study was registered by ICON Development Solutions, under registration number EudraCT 2012-000484-25.

### Participants

We recruited 24 healthy male adults, with normal body mass indexes (18–30 kg/m^2^) and normal laboratory health tests. Each committed to attend four visits experiencing pain stimuli on four different days within a two-month period. Participants were non-smokers or light smokers (up to 5 cigarettes or equivalent per day). We focused this study on males since resources were limited and we wanted to reduce gender-based interactions and effects, as well as avoid menstrual-cycle changes and their impact on pain and physiology, which is a complex topic of ongoing research (21–23). Properly controlling the complexity associated with the female physiology would require a larger and longer study, even if it results in the same measure working for women as what we study here for men. Informed consent was obtained before commencing the study.

### Pain-Elicitation: Cold Pressor and Capsaicin

#### The Cold Pressor Test

After preliminary equilibration of the hand temperature, and after alerting the participant that the process would start in 2 min, the participant was instructed to put one hand (the one without the palmar EDA sensor) into a cold-water bath (2°C) for 2 min whilst continually recording the pain intensity using the eVAS with the other hand. The right hand and left hand were used alternatingly on different visits. At the end of 2 min, the participant was instructed to remove his hand from the water bath. Pain was scored continuously using the eVAS starting at the time of the immersion and continued throughout the immersion.

#### The Intradermal Capsaicin Test

Participants were familiarized with pain evoked from 100 μg of capsaicin at the Training Visit. A single intradermal injection of capsaicin (100 μg) was made into the volar surface of the upper forearm (Manufacturer: ICON Development Solutions Manchester. Composition: 1 mg/ml capsaicin in 10% v/v ethanol, 7.5% v/v Tween 80 in 0.9% sodium chloride solution (100 μg/100 μL).) The injection of intradermal capsaicin was announced to the participant 5–8 min before the injection. The right arm and left arm were used alternatingly on different visits. Pain was scored continuously using the eVAS starting just prior to the intradermal injection and continued for 15 min after the injection.

### Pain-Measurement: eVAS and EDA

#### Recording of eVAS

Pain intensity was assessed using an eVAS with the left end (=0) being equivalent to “no pain” and the right end (=100) referring to “worst pain imaginable”. Participants were asked to evaluate pain intensity continuously by selecting the point on the eVAS that corresponds to the pain intensity they have at that moment in time. Participants were instructed to take both pain intensity and unpleasantness into account when scoring pain. While these can be considered two different dimensions, many studies show similar behavior of both dimensions during experimental pain, e.g., Duncan et al. (24), and efforts to distinguish them are ongoing (25) and not addressed in our study design.

#### EDA

EDA was measured electrically as skin conductance, using the Affectiva Q sensor, which measures skin conductance level (SCL) in microSiemens using 1 cm Ag-AgCl dry electrodes. Sampling rates were 8Hz. Each participant wore synchronized Q sensors on five different locations, left wrist (LW), right wrist (RW), left ankle (LA), right ankle (RA) and right or left palm (P). The palm side was alternated over the four visits and was worn on only one side because the cold pressor test required submersion of one hand into ice water. In the rest of this paper, only the data from the four limbs was used as the palm data was too often noisy from movement artifacts.

### Protocol

**Figure 1:**
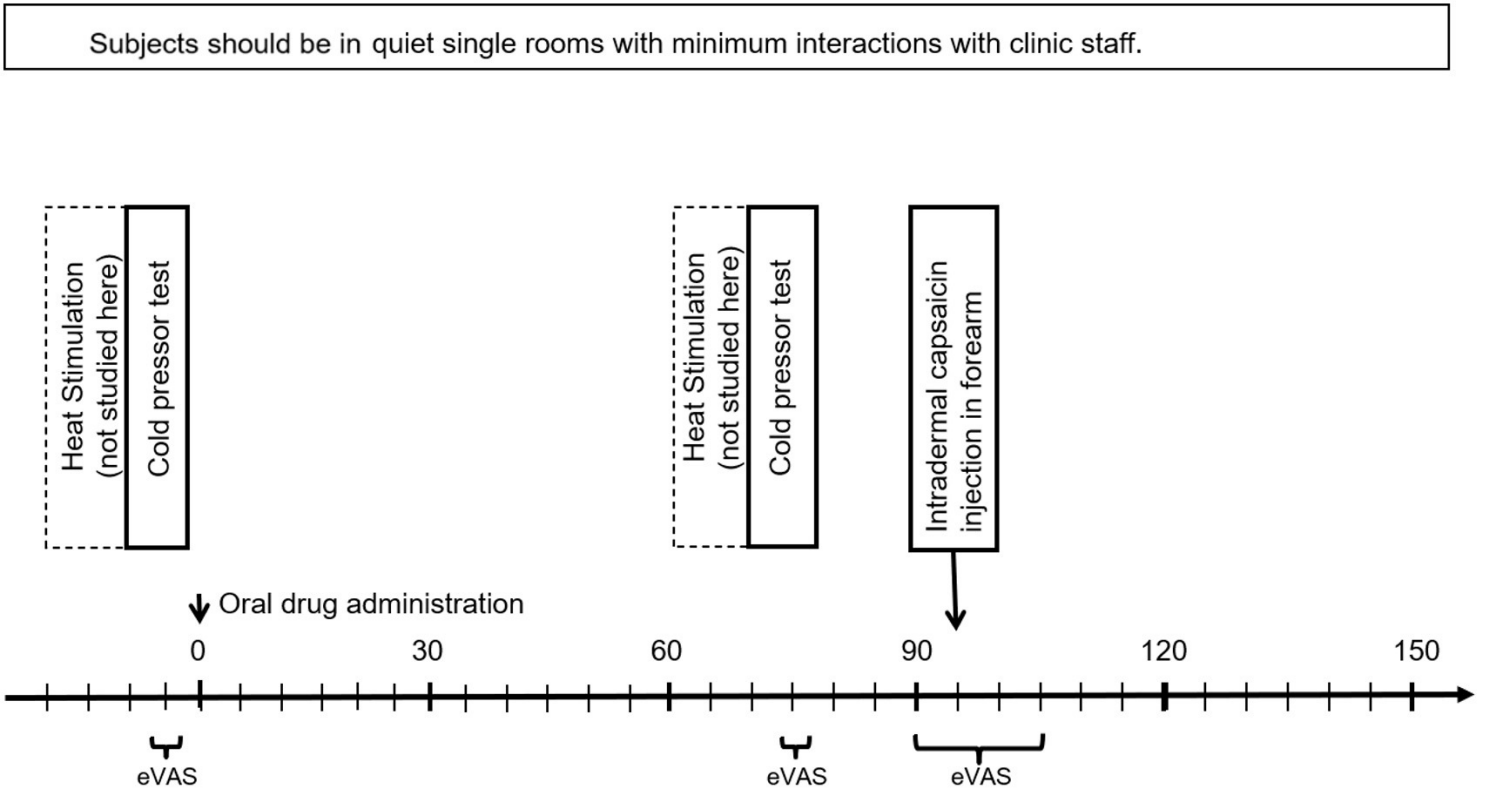
Study protocol, showing three time periods where eVAS was measured. EDA was measured for the entire session (~3 h).

The Protocol is illustrated in Figure 1. Each patient made four visits: An initial training visit (TV), followed by three treatment visits (Treatments A, B, and C) in randomized order. Treatments were applied double-blind to treatment condition, and all data analyses in this paper were conducted initially without the condition being revealed. Later, they were revealed to be: A=pregabalin, B=oxycodone, and C=placebo. All four visits had a similar structure: First the patient put on the five EDA sensors. Next, a baseline heat-pain stimulation on the non-dominant hand was performed (but is not analyzed in this work, as the timing of each part of the series of rapid stimulations was not reliably recorded for comparison to the EDA). Next, they experienced the cold pressor, while filling out eVAS continuously during the immersion. Then the treatment was applied in the form of an oral capsule, except during the first visit, the TV, when no treatment was made. Then, the patient rested for an hour, which allowed treatment A, B, or C to take effect. Next, they experienced again the same cold pressor test, while continuously reporting eVAS levels. Then, 10 minutes elapsed while they filled out the State Trait Anxiety Inventory (STAI) (26) (not analyzed here). Next, the capsaicin was administered while they continuously reported eVAS levels. Finally, the sensors were removed and the participant was dismissed.

Overall, this design enabled systematic examination of multiple important comparisons including: (1) measuring the same stimulus (cold pressor) before and after treatment (no treatment, placebo, oxycodone, or pregabalin) within the same day; and (2) measuring reported pain across different days (cold pressor and capsaicin) x (no treatment, placebo, oxycodone, pregabalin). Since a person's physiological patterns can vary a lot from day to day, it is important to see if the proposed eVAS-peak-anchored electrodermal measure shows consistent differentiation both across days as well as within days.

In an effort to mitigate the effects of anticipatory arousal, which is likely to increase with increasing uncertainty, the sequence of events was first shown to all participants up front during their first visit (TV), and then this same sequence of events was used in that visit and all subsequent visits. Only the treatment (blinded administration of A, B, or C) was randomized across the subsequent visits. All visits followed the same procedure to reduce the influence that uncertainty has on autonomic stress responses. Also, participants are given an indication 2 min before each cold pressor task that it is going to start in 2 min and similarly 5–8 min before the capsaicin injection, so that anticipatory effects and time periods are held as constant as possible across the procedures and days. This helps eliminate “surprise” effects on autonomic responses.

### Data Processing

ICON recruited 27 healthy adult male participants. Of these, three men dropped out of the study early and we received data sets for 24 participants x 4 sensors (LW, RW, LA, RA) x 4 visits = 368 sets of physiological responses. One participant's data had the wrong sampling rate for visit 1, no eVAS for visit 2, and no data for visit 3, so we dropped his data, leaving 23 sets.

Each file was visually inspected to confirm that the data record contained quality signals throughout the entire visit. Some files needed to be omitted due to bad data quality (malfunctioning sensor or sensor placed too loosely to record, causing visibly high levels of noise). Also, a total of 9 participants missed some visits or dropped out at some point after completing the training visit. Overall, 295 of the potential 368 files from the four limbs and 92 visits were obtained with high quality (80.2%). These 295 are distributed as: TV = 76 files, A = 73 files, B = 73 files, C = 73 files. All of these are used in the analyses that follow.

#### EDA Filtering, SCL Normalization, and Down-Sampling

Electrodermal activity can be divided into the “tonic component,” the slowly varying part of the signal usually referred to as skin conductance level (SCL), and the “phasic component,” the relatively fast changing peaks usually referred to as skin conductance responses (SCR's). The SCL is usually measured over intervals ranging from tens of seconds to hours, while SCR's are usually measured within 1–6 s after a discrete event.

Our analysis over the cold and capsaicin regions used SCL's derived as follows:

To separate the tonic from phasic EDA, a 5th order, zero-phase, lowpass Butterworth filter was applied to the raw skin conductance signal. The filter's cutoff frequency was set to 0.05 Hz as tonic activity is observed in 0–0.05 Hz. The SCL for each 1-min epoch was estimated using a 1-min wide centered moving average filter.

We compared data from multiple bodily locations and from multiple people over multiple days as baseline physiology can vary from day to day. We needed a robust way to make the data values comparable across all these files. Also, to accurately assess the changes in SCL after an analgesic, it is necessary to compare the SCL before and after the treatment on a common scale. We chose to use Z-score normalization before making all of these comparisons. To perform Z-score normalization, the (low-pass filtered) SCL for each file (one sensor, one day's session) was used to compute the mean and standard deviation for that session. Then the file's SCL was normalized by subtracting the mean value for the day's session and dividing it by the standard deviation for that session, such that the normalized SCL for the day has zero mean and unit standard deviation. Thus, the two cold and one capsaicin session for a person's visit were normalized using the same mean and standard deviation for that day.

The normalized SCL was subsequently analyzed over each of the regions I, II, and III for each cold and capsaicin segment.

#### Computation of Three-Region Measure

Pain is a mix of psychologically perceived phenomena and physically experienced phenomena (13). In this work, we combine these two components in a novel way, using two time points—the objectively measured onset time of the pain stimulus and the subjectively measured time of the peak of the self-reported eVAS data—to structure the analysis of the physiological EDA data into three regions of the pain experience. The result gives a measure that improves on eVAS by adding objective data yet incorporates a valuable aspect of the self-reported pain experience—its peak pain moment as perceived by the participant in pain.

**Figure 2:**
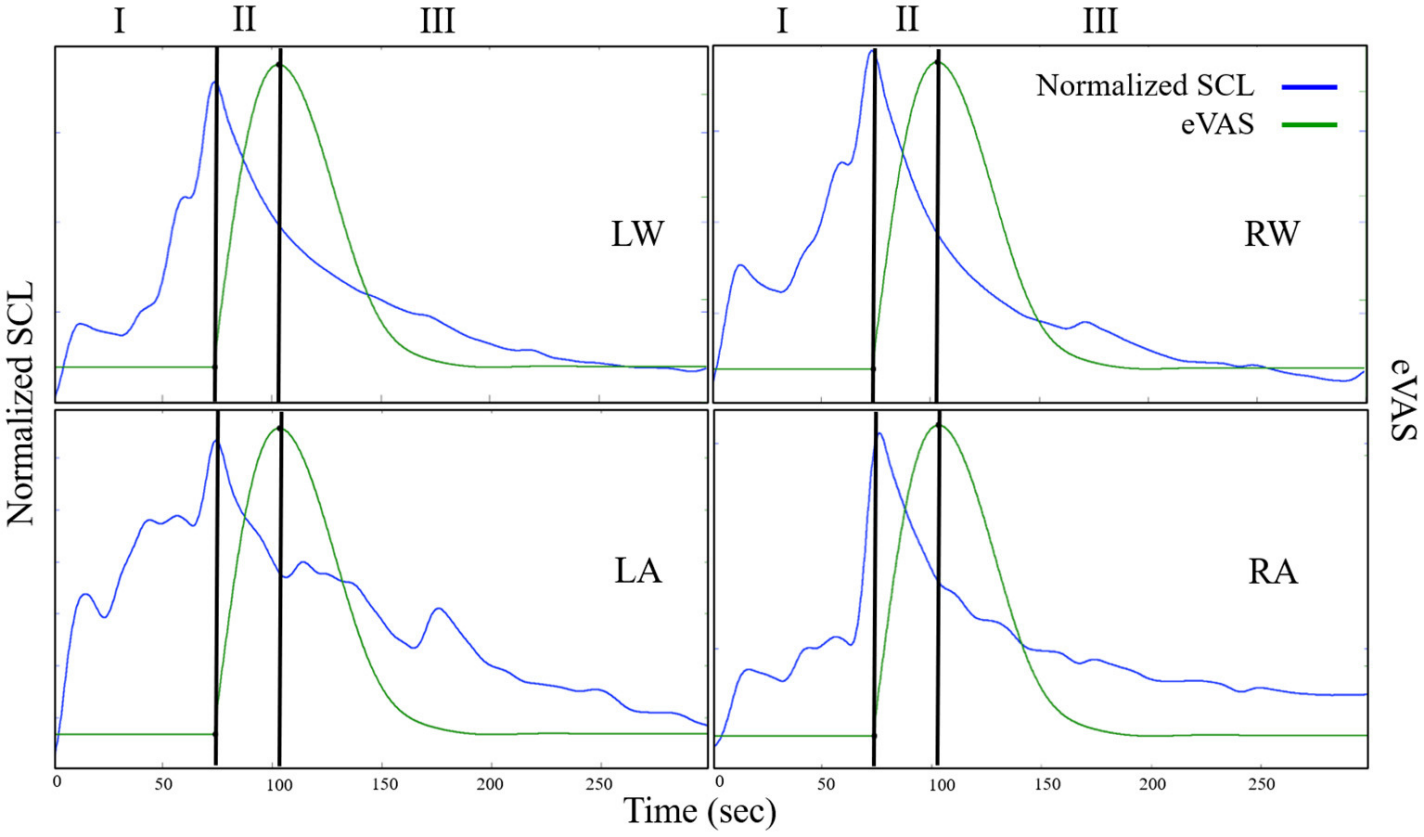
Examples from one person illustrating SCL (blue) and eVAS (green) during the Capsaicin segment for the left wrist (LW), right wrist (RW), left ankle (LA) and right ankle (RA). Region I starts when the person is told that the capsaicin treatment is next, and ends when the needle is inserted. Region II starts with needle insertion and continues until the person's self-reported eVAS reaches a peak. Region III is measured from the peak eVAS until 15 min after the onset of the capsaicin treatment. Region III for Capsaicin usually contains decreasing values of SCL, as in the examples shown here.

Here is how the three-region method works (See Figure 2): Using eVAS data and timing information of when the person was warned of the impending cold or capsaicin stimulus, we define three non-overlapping regions. These three regions separately quantify three regions of the pain response: I. Anticipation, II. Response, and III. Recovery.

##### Region I = Pain Anticipation

For the cold stimulus, participants were warned approximately 2 min before the cold pressor test. For the capsaicin stimulus, the warning period was from 5 to 8 min. We define Region I, “the anticipatory period”, to be the region of time from the onset of the warning to the onset of the pain stimulus. We expect that SCL during this region is affected more by anticipatory anxiety than by physical pain. It is important to include responses during this region because sometimes people appear to actually experience pain before the stimulus touches them: For example, a child might jerk back and scream with “pain” before a needle touches them, and adults sometimes exhibit a facial grimace as if in pain before the onset of actual sensory pain. Thus, we include Region I, the subjective pain anticipatory experience, as part of the pain experience. The eVAS was not reported during region I so we cannot compare physiology with eVAS in that region. However, SCL is hypothesized to rise with anticipation, uncertainty, and anxiety, and our study data confirm that the SCL usually rises during Region 1, even sometimes taking on high values here.

##### Region II = Pain Response (Rising From Onset to Peak)

Region II is defined as the region of time that begins with the onset of the pain stimulus and ends when the person reports their peak pain level. In this study, the cold stimulus begins when the hand is placed in the ice water, and the capsaicin stimulus begins when the needle is inserted. The participant begins to report eVAS at this onset moment. Region II spans the time from the start of the pain stimulus and start of the eVAS recording to the peak reported eVAS level. The timing of this peak is clearly visible for capsaicin, which has eVAS that tends to follow the shape shown in Figure 2 (green line = eVAS, blue line = SCL).

**Figure 3:**
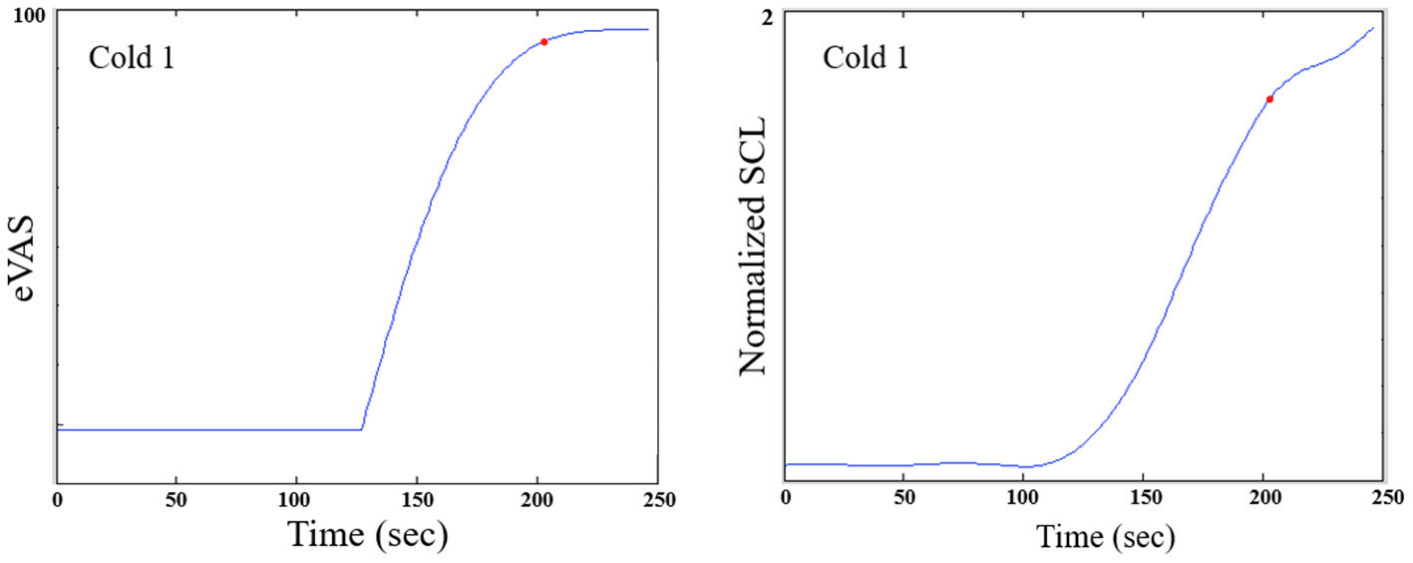
For the cold pressor, the eVAS tends to grow monotonically throughout the task. Thus, a region defining Region III strictly as following the “peak" could be of zero duration. We thus define a function of the peak as the point at which the eVAS does not change > 0.005. The red dot shown above marks the onset of Region III for this participant. Typically, the SCL does not drop during Region III of the Cold pressor test; thus, we measure Region III over a fixed 2-min duration.

For cold pressor, we compute the peak location differently, as the eVAS often climbs monotonically and doesn't peak until the 2-min cold pressor test ends (See Figure 3). If we counted the peak as the right-most point, then we would often have just Region II and no Region III. We think it is valuable, even though the hand is still immersed, to examine this later portion of the cold pressor task where the eVAS tends to “level off” separately from the first portion of the immersion, where the eVAS typically climbs fast. Thus, for cold pressor pain we define the peak to occur at the time that the eVAS levels off–specifically, where it ceases to go up more than 0.005 units or 99.99% of the maximum value.

##### Region III = Pain Recovery (Sustain or Decay)

In this study, for both cold pressor and capsaicin, Region III is defined to begin at the peak identified in the eVAS. For cold pressor, Region III is measured until the cold pressor is ended (2 min from cold pressor onset), while for capsaicin, Region III is measured until 15 min following the onset of the capsaicin stimulus. For cold pain, this region is where the eVAS is usually leveling off–pain is “sustained.” For capsaicin pain, this region tends to be where the eVAS values “decay” as the person is in recovery from the initial capsaicin injection.

Using these pain regions, I, II, and III, defined by the times of announcement of the stimulus, the onset of the pain stimulus, and the time of the patient-reported eVAS peak, we next examine how an objective measure–the normalized, low-pass filtered SCL–changes both within each region and across the regions for each type of visit and each type of treatment. In particular, we wish to evaluate if the new method presented here, anchoring physiology relative to these three regions, is useful for more objectively measuring pain and for measuring the efficacy of active vs. placebo treatments.

Below we examine the performance across three pain-stimuli events: Cold1 (cold pressor applied before the treatment pill was consumed), Cold2 (cold pressor applied more than an hour after the pill was consumed), and Capsaicin (after the Cold2) during each of four visits made by each participant: Training visit (TV), Treatment A, Treatment B, and Treatment C. The oral treatment was administered on visits A, B, and C between the first and second half of the session in a double-blind way by a staff person who came in the room to give them the pill and otherwise did not observe the patients during the trials. Thus, the analgesic is given time to take effect before Cold2 and Capsaicin on visits A and B, while the placebo is given for visit C. The visit ordering was randomized across the patients, with the exception that no treatment is given during the first visit, TV.

## Results

### Cold Pressor Pain

For each visit, a participant experiences two cold pressor tests: Cold1 (before the pill is given) and Cold2 (after the pill is given). On the training visit, there is no pill given but the patient rests for the hour between the two cold pressor tests. Our hypothesis is that participants will experience significantly less pain during Cold2 than during Cold1 when the treatment contains an analgesic (A or B) and the difference in pain between Cold2 and Cold1 will be small during C (placebo) and insignificant during TV. We also predict that the response to Cold1 will be highest on the first visit (TV) because of the extra anxiety and uncertainty associated with the novel pain experience.

**Figure 4:**
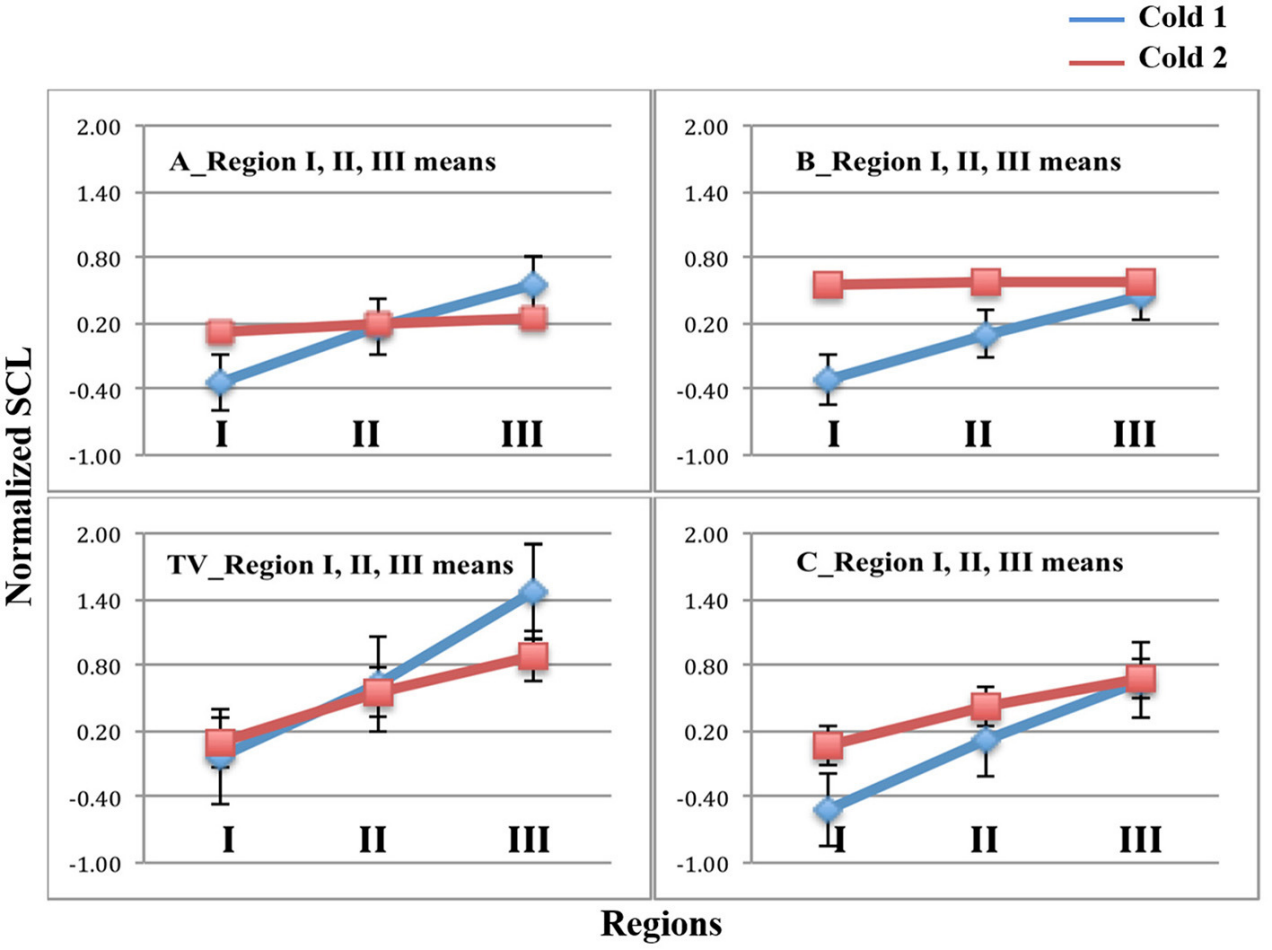
Mean and standard error bars of normalized SCL in Regions I, II, and III. Blue = Cold1. Red = Cold2. Data are from the four limb sensors for n = 23 participants. Analgesics were applied in A and B, and are associated with a lack of increase across the Cold2 pressor.

In Figure 4, we see the results of the analyses applied to the full set of data. The bars indicate standard errors. The data from sensors on the LA, RA, LW, and RW were compared for *n* = 23 participants. The blue lines represent the mean normalized SCL values of regions I, II, and III during Cold1. As hypothesized, it is seen that, the Cold1 lines (blue) climb in value during all four visits, TV, A, B, and C, i.e., the means increased steadily through all three regions for all the Cold 1 episodes, as there is no treatment present during any day's Cold1 experience. The largest normalized SCL increase is observed during TV's Cold1. While this is not a finding central to our hypotheses, it was expected nonetheless: We have usually seen in studies with SCL that greater uncertainty is associated with higher SCL. Given this was the first visit, and the first experience of an unknown amount of (untreated) pain, it is expected that this visit would have had both the highest uncertainty and the highest increase in SCL. It is one of the reasons we designed the study to have a TV and also a placebo control, so that benefits of the new method are not overestimated by this “first visit” effect.

In these same figures, the red lines represent the mean normalized SCL during the second experience of Cold pressor pain each day, Cold2. We see for TV's untreated and C's placebo treatment that the SCL climbed over time, similar to all the visits in Cold1. For analgesic treatments A and B, however, the objective SCL shows that even if the overall level was a little higher at the moment of starting the pain segment (e.g., from hypothesized higher sweating when taking analgesic B), the relative increases that usually happen from the pain onset to peak, and beyond, were clearly attenuated by both analgesics (Note that the stderr bars are too small to be seen graphically in the plots for five of the measurements shown).

**Figure 5:**
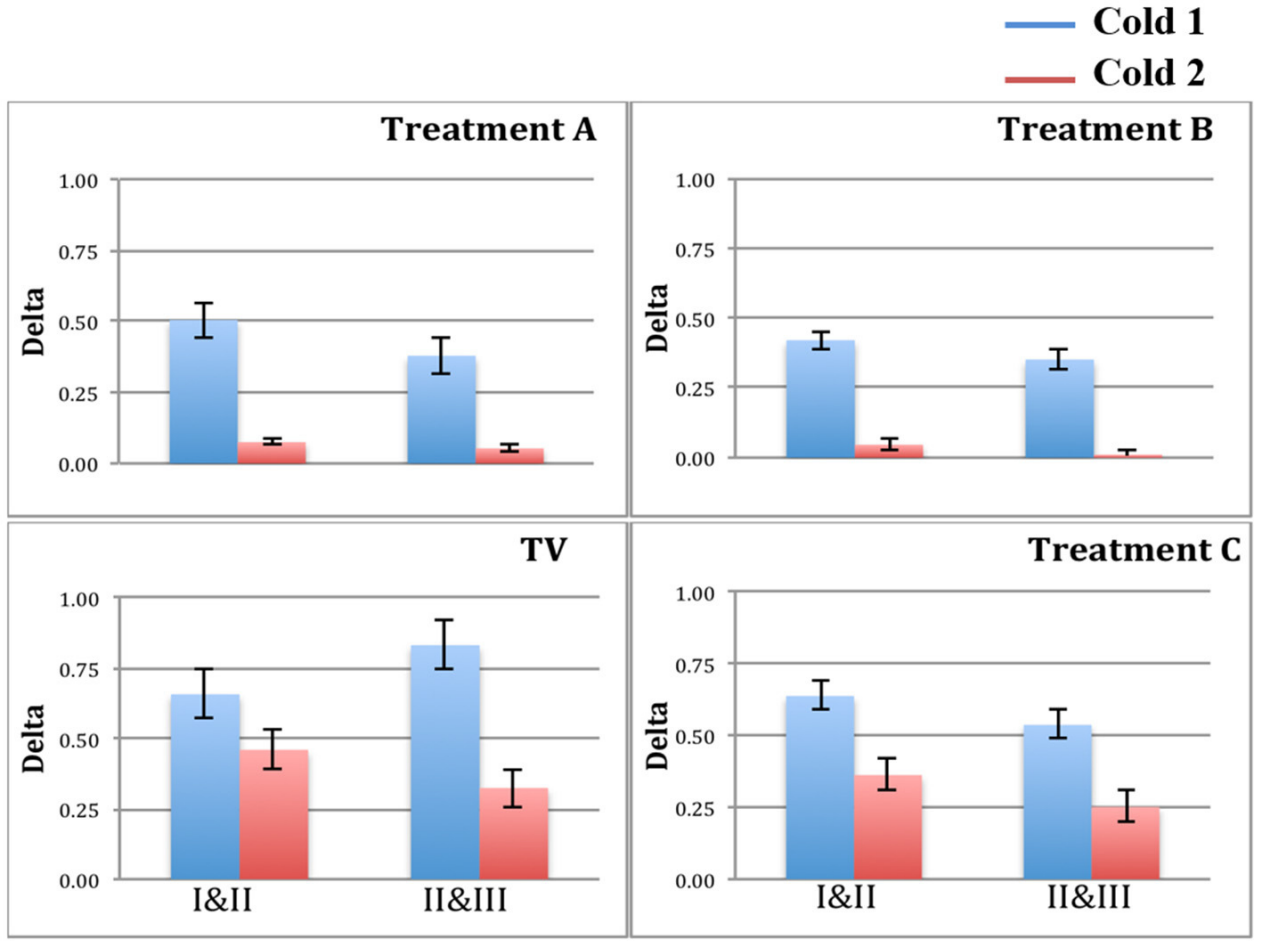
Blue bars = delta values between regions for Cold1. Red bars = delta values between regions for Cold2. Treatments A and B show a significant reduction in the deltas, as hypothesized for these two analgesics.

Figure 5 plots the two deltas (difference in mean normalized SCL) between adjacent regions of the pain experiences. As predicted, since there is never an analgesic at the time of Cold1, the deltas for Cold1 (blue bars) are significantly larger than the deltas for Cold2 (red bars). The statistical significances of the measures shown in Figure 5 are computed and shown in Table 1. We performed a Wilcoxon Rank Sum test on the deltas between regions I and II, and regions II and III, during Cold1 against the respective deltas during Cold2 (This test was chosen because the deltas do not have a normal distribution). We compared the data from 23 participants and for all the limb sensor locations together for each treatment. We see all hypotheses confirmed: for TV there is no significant difference in the SCL changes during the first and second cold pressor tests, which is as expected given no treatment is given. As hypothesized for analgesic Treatments A and B, there was a significant difference before and after the treatment was given. This difference is consistent with the hypothesized significant reduction in pain expected following treatment A or B, both of which are well-known effective analgesics.

**Table 1:** Testing for statistically significant changes in normalized SCL between adjacent regions during Cold1 (before treatment) and Cold2 (after treatment).

**Δ**	**TV**	**TV**	**A**	**A**	**B**	**B**	**C**	**C**
	**I and II**	**II and III**	**I and II**	**II and III**	**I and II**	**II and III**	**I and II**	**II and III**
h	0	0	1	1	1	1	1	0
p	0.355	0.151	0.000	0.000	0.000	0.000	0.023	0.065
N	75	75	76	76	73	73	73	73

*One-tailed Wilcoxon rank sum test (testing if delta Cold2 < delta Cold1 and assigning h = 1 if this is true). The significant effect of the analgesic is confirmed for treatments A and B, while a significant effect is also seen only at the start of the placebo C*.

We hypothesized a small reduction in pain in the placebo condition, C, which we also found using the new measure. Interestingly, in condition C, we see a small but statistically significant difference involving the anticipatory period, but not during regions II & III once the pain has become established. Thus, the placebo shows a transient impact on the onset-to-peak pain but no impact on the sustained pain. This is an interesting aspect of the three-region method: It is deliberately separating out the anticipatory region, which may be the region most impacted by cognitive and affective beliefs, such as belief about the helpfulness of a placebo.

In Table 1, h = 1 is used to designate when the changes in SCL between regions I and II, and between regions II and III, were significantly lower. We see that h=1 occurs after the drug for analgesic treatments (in Cold2 for both A and B). For the placebo treatment C, the reduction from region I to II for Cold2 can be interpreted as a transient placebo effect that reduced the pain response during the anticipatory period preceding the stimulus onset. The reduction did not occur from region II to region III for the placebo.

We performed another comparison to further test the significance of the new three-region measure by comparing pain responses under a treatment (deltas between regions I and II and between regions II and III) for treatments A, B, and C, with pain responses during the training visit (no treatment during TV). Passing these tests is more challenging since they occur across different days. An objective physiology-based measure that shows reliable results across cold-pain experiences on different days is less likely than one that shows results only within the same visit's Cold1 vs. Cold2 comparisons because people tend to have different physiology from day to day due to hydration, mood, stress, and other natural variables. Further, mood or stress effects can also bias each person's self-reported eVAS range from day to day.

As seen in Table 2, looking across the multiple days of visits, the new measure's deltas for treatment A and treatment B were still significantly different than were the deltas during the TV and treatment C (placebo) for Cold2. Comparisons were made with a 1-tailed Wilcoxon test. These results show that the median changes in SCL from region I to II and from region II to III during Cold2 (after the drug) for treatment A and treatment B were significantly smaller, as hypothesized, than the median changes in SCL during Cold2 for TV. Thus, the measure shows that the analgesics result in a significantly reduced pain experience, unlike the placebo and no-treatment conditions, and this effect captured by the new measure is robust across different visits.

**Table 2:** Testing for statistically significant changes in normalized SCL for Cold1 (before treatment) and Cold2 (after treatment) across sessions on different days.

**Δ**	**TV vs. A**	**TV vs. A**	**TV vs. A**	**TV vs. A**
	**Cold1_I and II**	**Cold1_II and III**	**Cold2_I and II**	**Cold2_II and III**
h	0	0	1	1
p	0.485	0.436	0.000	0.001
N	75 vs. 76	75 vs. 76	75 vs. 76	75 vs. 76
**Δ**	**TV vs. B**	**TV vs. B**	**TV vs. B**	**TV vs. B**
	**Cold1_I and II**	**Cold1_II and III**	**Cold2_I and II**	**Cold2_II and III**
h	0	0	1	1
p	0.213	0.142	0.000	0.000
N	75 vs. 73	75 vs. 73	75 vs. 73	75 vs. 73
**Δ**	**TV vs. C**	**TV vs. C**	**TV vs. C**	**TV vs. C**
	**Cold1_I and II**	**Cold1_II and III**	**Cold2_I and II**	**Cold2_II and III**
h	0	0	0	0
p	0.545	0.788	0.651	0.659
N	75 vs. 73	75 vs. 73	75 vs. 73	75 vs. 73

*Comparison is made between the first visit, TV (no treatment for Cold1 or Cold2), and later visits A, B, C. As desired in a measure, all of the no-treatment conditions do not differ over time. The effect of the analgesic is significant only in the diminished responses of SCL to Cold2 in conditions A and B (and not in Cold1 conditions or in placebo C)*.

These tests illustrate several strengths of the proposed new measure: A valid objective pain measure should show that treatments A and B reduce pain compared to placebo treatment C, and compared to no treatment. The results in Table 2 confirm that the new measure shows these statistically significant reductions for Cold2. We see the significant effect of comparing analgesic conditions, A and B, to non-analgesic condition TV, and the non-significant effect of C's placebo compared to TV. Moreover, the new measure's significant differences cannot be attributed simply to day differences, as the study further confirms the presence of no such difference across the days during Cold1, before the analgesics are applied (where all h = 0).

### Capsaicin Pain

For each visit, each participant experiences one inoculation of Capsaicin to elicit pain. We make similar tests for the Capsaicin pain model. The big difference in these tests is that now we have only one inoculation per visit in the second half of each visit, so we cannot compare pre- and post-drug within the same day's visit. Instead, we must evaluate the harder challenge of comparing across visits that have analgesics (A, B) and that don't have analgesics (TV, C), even though these occur on different days. Thus, to find a reliable, repeatable result for Capsaicin pain is a greater test of the new measure's robustness than when the measures are made within the same day's session.

**Figure 6:**
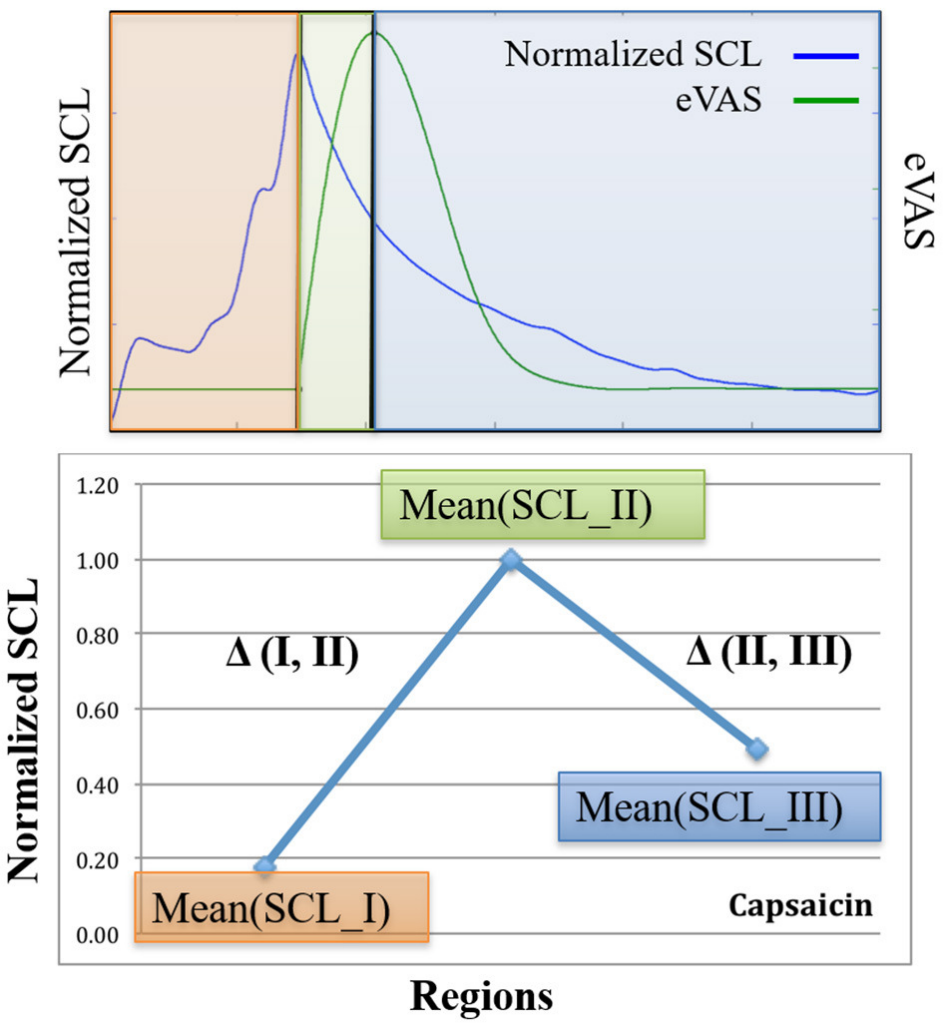
Notation for Capsaicin regions and deltas.

We first characterize the changing pattern of mean SCL across Capsaicin regions I, II, and III, as this is a different kind of pain than cold pressor pain. The notation we use for Capsaicin is described in Figure 6, where we again denote the three regions relative to the time of onset of the needle (pain stimulus) and to the (subjective) eVAS-reported peak pain. Again, we show the eVAS in green, and we use its peak to separate regions II and III. The SCL is shown in blue, having an earlier peak at the end of the anticipatory region, the moment when the needle is applied. The first time we saw this “anticipatory” peak preceding the actual reported peak-pain we were surprised (This occurred in a prior pilot study with flu-shot data, where it occurred the moment before the needle was inserted). We find in this clinical trial data that such a peak sometimes occurs as in the example shown here, and sometimes occurs closer to the self-reported peak pain. This phenomenon is another reason to explicitly measure Region I.

**Figure 7:**
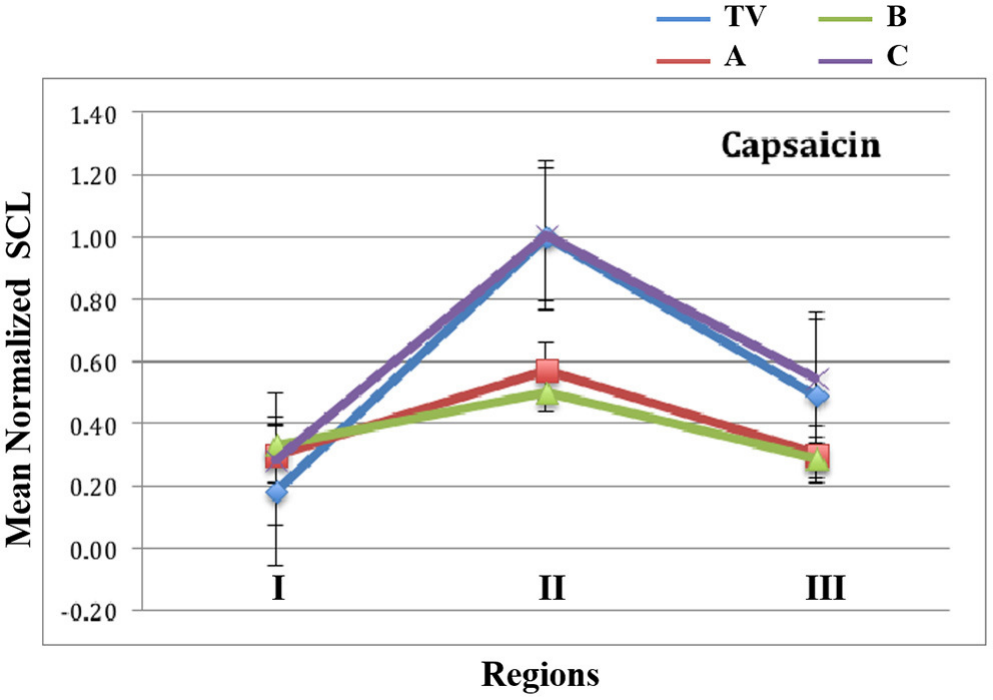
Mean values of normalized SCL within regions I, II, and III for capsaicin. TV = blue, C = Purple, and the analgesics A = red, B = green. Data combine four limb locations from n=23 participants; bars are stderr.

In Figure 7, we show the mean normalized SCL for all four types of visits, during each of the three regions of the Capsaicin experience. First, we see a general arc across all the visits TV (blue), A (red), B (green) and C (purple): The anticipatory period is relatively low in all visits. In the middle, we see that the peak pain eVAS period is also in the region usually having the peak SCL. This property of this measure is seen to be robust for all four types of visits. Finally, the slow recovery of eVAS is similar to that of the SCL during Region III.

The capsaicin pain experience is divided into three regions: the anticipation of pain just before the injection, the needle pain with the injection and its feeling of pain increasing to a peak value, followed by the feeling of the burning wearing off slowly as the peak pain subsides.

As hypothesized, the proposed new measure shows that the arc of the three-region response is less severe for the two analgesic conditions A and B than for the non-analgesic TV and C conditions.

We examine the statistical significance of the measure by comparing the mean values of EDA in regions I and II and in regions II and III of treatments A, B, and C with those of the training visit TV. We apply the Wilcoxon rank sum test to examine the mean values within each region, across the conditions (Table 3).

**Table 3:** Capsaicin pain: comparisons of normalized mean SCL in treatments A, B, and C vs. the training visit, TV, within each of regions I, II, III.

	**TV vs. A**		
	**I**	**II**	**III**
h	0	1	1
p	0.409	0.000	0.041
N	75 vs. 76	75 vs. 76	75 vs. 76
**TV vs. B**				
	I	II	III
h	0	1	1
p	0.109	0.000	0.021
N	75 vs. 73	75 vs. 73	75 vs. 73
**TV vs. C**				
	I	II	III
h	0	0	0
p	0.110	0.323	0.478
N	75 vs. 73	75 vs. 73	75 vs. 73

*We see the hypothesized reduction of pain confirmed in regions II and III of the analgesic treatments A and B (h=1 for both). We also see the “no difference” hypothesis confirmed for the placebo treatment C, and for all anticipatory periods (before the needle is inserted)*.

Results show that the mean values of normalized SCL for region II are significantly different between A and TV, and between B and TV, and *not* between C and TV. These results are all in the hypothesized direction: The analgesics reduce the pain response more than placebo, which reduces it more than no treatment.

Importantly, the changes in our new measure are not due to an “overall reduction in SCL” from the analgesic because we confirm (Table 3) that the SCL is not different in Region I, before the onset of the pain stimulus, even though all treatments had been given more than 90 min before this time.

Note that these statistically significant effects, for both the capsaicin and the cold pressor pain models, were found before the team doing the data analysis was unblinded to conditions A, B, and C.

## Discussion

This paper presents a novel measure of characterizing pain response based on objectively identifying the time of onset of a pain stimulus, subjectively identifying the peak-pain moment (from the numerical peak of a self-reported eVAS), and then quantifying physiological changes in the three regions delineated by these two time points. The resulting quantitative measures are shown to provide statistically significant discrimination validating the effectiveness of well-known analgesics compared to placebo and no-treatment.

Does the new method work better than self-reported eVAS data alone, and if so, when might it replace it? Before showing this quantitative comparison, it is worth noting some of the features of traditional psychophysical methods of pain assessment, which request a report of subjective pain experience using either one-dimensional pain scales (like eVAS) or multidimensional pain scales; for a more complete picture of self-reported evoked pain response, various assessments must be used (6, 27–30). Different aspects of pain response such as psychological distress or anticipation, pain intensity, and pain recovery interact in complex ways to determine the perception and experience of pain (31–33). The proposed new three-region model summarizes these complex interactions quantitatively with three physiological values that capture meaningful differences in pain level across treatments both within a day and across days; however, the topic of how the measures of the three regions map to the many subjective aspects of pain, and their assessments by multidimensional pain scales, is not currently captured by the method in this paper. These topics remain a challenge for future studies.

**Figure 8:**
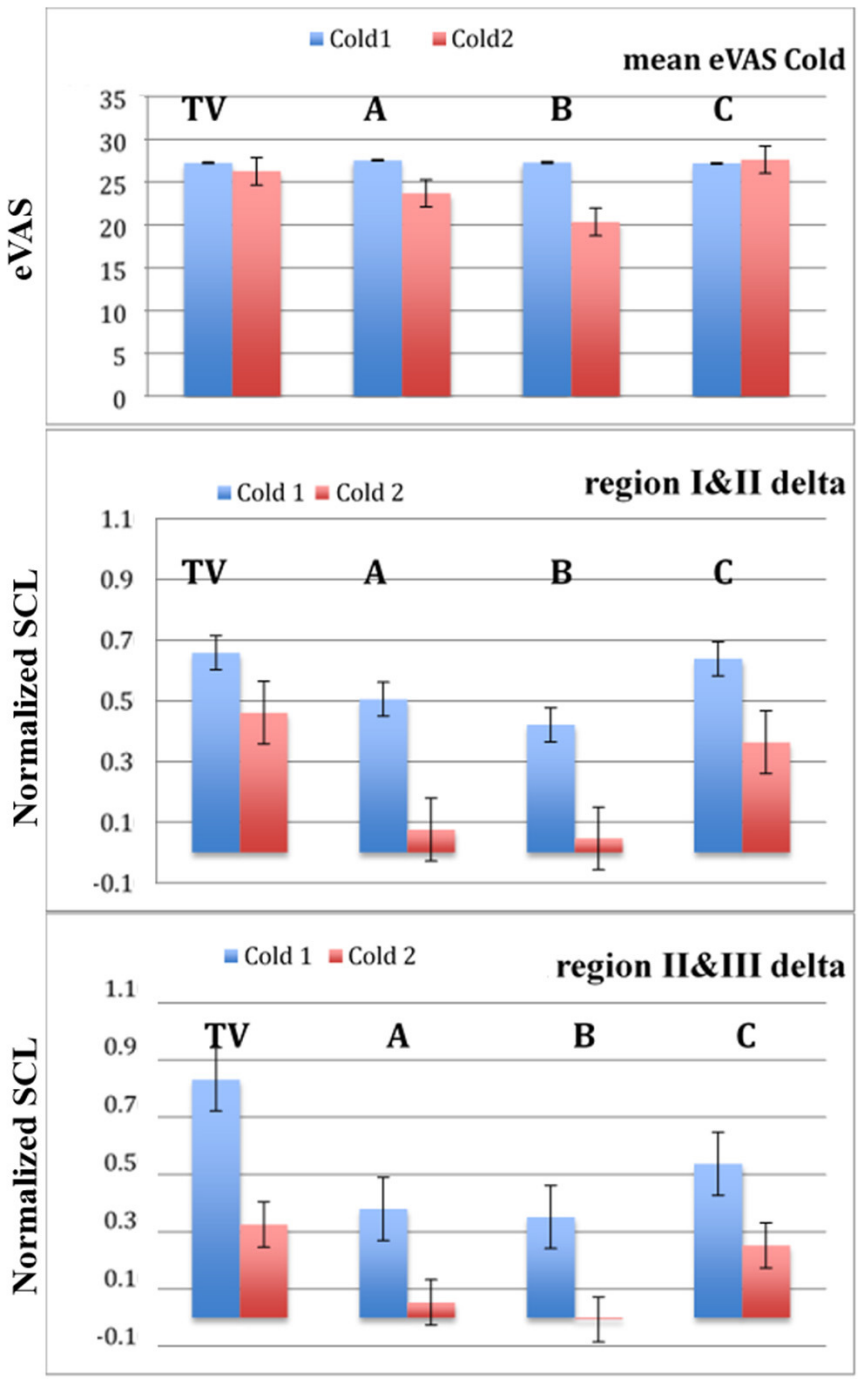
The mean values of eVAS alone do not successfully discriminate both effective analgesics. The deltas comparing normalized SCL values across adjacent regions are more discriminatory. Cold 1 = blue, Cold2 = red, n = 23 participants. Bars are stderr.

The new method adds some complexity to eVAS: eVAS is only one modality, while combining it with EDA integrates a second. Thus, we directly test: Is the new combined EDA + eVAS method performing objectively better than using only eVAS across this data set? The answer is yes, as seen in Figure 8 where we show the mean eVAS values across conditions TV, A, B, and C for Cold1 vs. Cold2, and Tables 4, 5 where statistical significance comparisons are made. We also tested the max eVAS values and the area under the curve of the eVAS, and the results were similar, with the only case of statistically significant discrimination occurring with eVAS and treatment B in the case of Cold pain, and with no significant discrimination with the Capsaicin model using eVAS alone. Another eVAS measure we tested was the time from the start of the stimulus to the max eVAS, which was found to not differ significantly across the visits for a given pain model. While this means that it fails as an eVAS measure at discriminating treatments that reduce pain, it does add strength to its use in our proposed new measure for defining Region II's endpoint, as it is stable across visits and across treatments. Thus, the value a person gives with the eVAS, used alone, fails to discriminate any pain reduction of using pregabalin for the more than 70 visits where eVAS measures compared Cold1 to Cold2, the latter after the treatment was given, and also fails to discriminate any pain reduction of either treatment with the Capsaicin model.

**Table 4:** Using eVAS, there is no difference in pain response for analgesic A and we see only a marginal difference for analgesic B.

**Test**	**TV-A-Cold1**	**TV-A-Cold2**	**TV-B-Cold1**	**TV-B-Cold2**	**TV-C-Cold1**	**TV-C-Cold2**
h	0	0	0	1	0	0
p	0.95	0.56	1.00	0.05	0.95	0.56
N	75 vs. 76	75 vs. 76	75 vs. 73	75 vs. 73	75 vs. 73	75 vs. 73

**Table 5:** Using only mean eVAS, the reported pain comparison for Cold 1 vs. Cold 2 differs significantly only for analgesic B.

**Δ**	**TV**	**A**	**B**	**C**
h	0	0	1	0
p	0.792	0.392	0.031	0.897
N	23	23	23	23

A limit of using only eVAS is also seen in the marginally significant difference found between TV and analgesic B (oxycodone) across visits when all measures are based on using only eVAS (Table 5). However, the three-region EDA + eVAS measure clearly distinguished both visits A and B from visits C and TV. Thus, the novel method outperforms traditional eVAS in a randomized control trial evaluating the cold-pressor model of deep pain. The new model is specific to pain (using eVAS to anchor the peak moment of pain) while being more discriminative than eVAS, even with a relatively small number of participants.

Note that it is possible that with a much larger number of participants, the difference between TV and A may eventually become significant when using only eVAS, as might at the same time the difference between TV and C. However, adding more patients adds substantial trial costs, and it requires inflicting pain on a lot more people. If the difference (Cold2 vs. Cold1) using the analgesic with a larger number of participants becomes significant, yet no greater than placebo's significance, then the drug will not be deemed effective. In contrast, the proposed new pain measure is significant in its discriminatory ability when using a small number of participants; thus, it may reduce both clinical trial costs and the ethical costs of inflicting pain on larger numbers of people.

EDA is traditionally recognized as responding to pain, but not specifically to only pain: It usually increases when the sympathetic nervous system is activated, with the fight or flight response, as well as with uncertainty and anticipation (16). Thus, an increase in EDA is usually expected with both anticipation of and experience of painful experiences. Using direct brain stimulation, researchers have shown that EDA is activated ipsilaterally by stimulation of the amygdala, anterior and posterior hippocampus, and anterior cingulate (34), key regions involved in processing pain, emotion, and anxiety. Thus, the EDA measure in general will be sensitive to pain, changing when pain happens; however, it is not specific to only pain; for example, a significant increase in EDA may occur with brain activity during and soon after a grand mal seizure (35); also, it has been observed to be elevated at the time of death in the minutes following a grand mal seizure (36).

Our work here addresses the problem of specificity in several ways. First, like with early work showing that skin conductance responses reflect infant responses to painful heel sticks (37), we measure the level of pain objectively in a situation known to cause experience of pain, as would be expected in a clinical study, hospital, or recovery room, where contextual factors that might influence the pain measure are both observable and controllable. Second, and novel to our work, we specifically anchor the regions to-be-quantified by using the time point where the person explicitly marks their (subjective) peak pain experience. Third, the way that we process the EDA data within the three regions removes effects likely to be influenced by the environment or other day-to-day varying influences: This was shown in our study design requiring visits on different days, likely to span different conditions of hydration, heat, and humidity. Finally, the way we designed the study with a training visit (TV) helped reduce influences due to study-specific effects on emotions that can be caused, for example, by the first visit's arousal where a patient experiences uncertainty and possible fear or anxiety related to the experimental conditions such as “What are they going to do to me next?” or “How badly will it hurt?” The resulting method thus works specifically for pain, as demonstrated not only with one pain model, but with two very different pain models.

We presented a novel method for improving upon a traditionally subjective method of pain measurement by defining three regions of the pain experience, anchoring these specifically to a patient's personalized ‘peak' pain moment, quantifying objective autonomic data for each of the regions, and testing the discriminability of the method over 92 patient visits, including four conditions—two analgesics, a placebo, and a “no treatment” condition—within a randomized control trial. The method uses one piece of information from eVAS—the timing of its peak self-reported pain—but otherwise does not use any of the actual values from the subjectively-reported scale.

One of the interesting findings in this study was how the three-region measure gives insight into physiological changes occurring with the placebo condition, C. In all of the comparisons using the three-region measure, the placebo response was found to lie between that of the no-treatment training visit (TV) and the analgesic treatments (A and B), confirming well-known expectations about placebo effects. This finding is based quantitatively on the objective data from the physiology. Interestingly, when we separately examined the three regions of the pain response, the placebo condition was seen to have its largest affect during the anticipatory period, with smaller effects during regions II and III, once the pain became established. This finding suggests that for those who continue to use only eVAS to measure pain, they may find a different significance level simply by asking patients to report their pain at a different time. We hypothesize that the placebo effect has a different temporal trajectory than the analgesic effect. We suggest that future work examine its dynamics, which could have significant bearing on clinical comparisons, allowing the statistical significance of clinical findings that rely upon eVAS to be manipulated by adapting the timing of when pain is assessed. Methodologically, this timing is an important piece of information, and we suggest it should be reported in future pain study designs to add extra integrity to the design. Note that when today's methods use a “one value” rating of pain for the entire experience (corresponding to our regions II and III), then it will obscure this information (e.g., Figure 8's average eVAS ratings).

Our method was shown to appropriately address the concern that an opioid (oxycodone, treatment B) causes higher sweating than another analgesic (pregabalin, treatment A) and increased sweating might interfere with a method based on SCL (38). We thought it was especially important to test this effect since we measure SCL on the wrists and lower legs, and these sites are sometimes (without evidence that we have seen) claimed as being more thermoregulatory than emotional, even though there is plenty of evidence, across many types of studies, that non-thermoregulatory events, such as those due to changes in neurological (e.g., seizures and sleep stages) and cognitive-affective states result in changes in SCL at these limb locations (16, 35, 36, 39–42). We thus examined the mean values of SCL across the four limbs during Cold 2 to see if there is more overall response with treatment B. As we saw previously in Figure 4, Treatment B's mean normalized SCL has a higher value for Cold2 in region I compared to A, C, and TV. This region is immediately after the treatment is given orally (and allowed to take effect during rest) so we do see increased sweating in the baseline at the start of Cold2. However, our measure considers the change in SCL from region I to II, and from II to III, both of which remain low.

**Figure 9:**
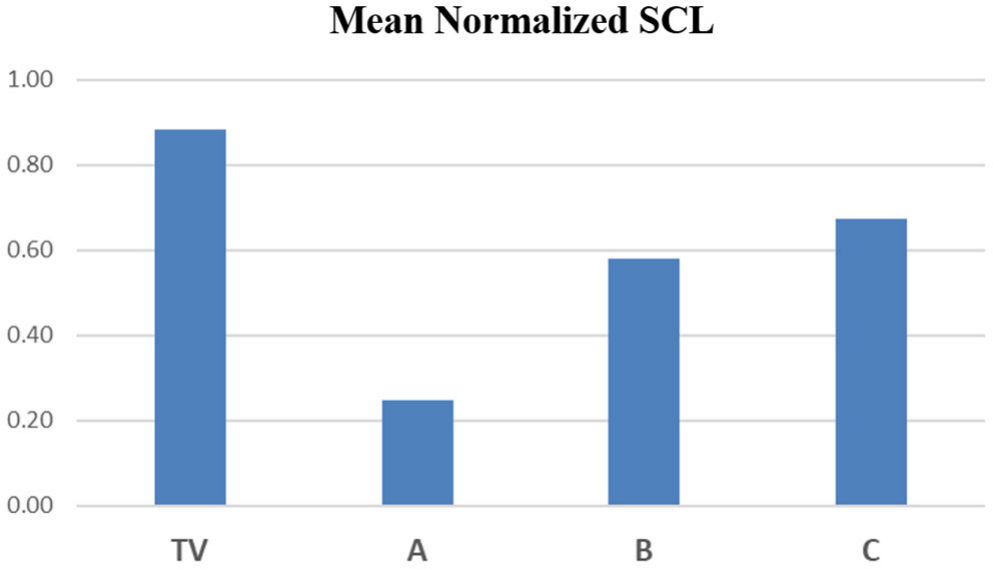
Mean values of normalized SCL from the four limb sensors during Cold2's Region III.

In Figure 9, we continue this examination by plotting the mean normalized SCL values from the four limb sensors during Cold2 region III, across all participants. This region corresponds to the highest SCL in the non-analgesic visits (TV followed by C = placebo) and is reduced significantly for analgesic A. While analgesic B shows values that are reduced from those in the TV and C conditions, we do indeed see higher SCL on average in B than in A, which is consistent with the reports of increased patient sweating with this drug. However, the mitigating effect of the analgesic on the three-region physiological measure is still statistically significant despite the increased sweating. In short, the proposed three-region measure of pain appears to change in a way consistent with reduction in pain, not simply with the amount of sweating. It is robust at discriminating pain relief even given the effects of increased sweating from an opioid (oxycodone).

Returning to Figure 4, not only is the mean SCL higher for Cold2 treatment B in region I, but also it appears to be relatively higher than Cold1 in all of the non-first visits (A, B, and C) in region I. While the reason it is elevated for the opioid condition B is described above, it is interesting to consider what might affect this region I disparity in the other cases. While we cannot be sure of the cause without running a future causal experiment design, we consider some likely contributing factors here: (i) The region I for Cold1 is lower on repeated visits, which we expected because the Cold1 experience is unchanged from the first visit, and thus uncertainty about it is reduced. (SCL tends to increase with increasing uncertainty). (ii) While the repeated visits repeat the entire protocol, there is a novelty at the start of Cold2 during the visits A, B, and C. In these visits, participants have just consumed an unknown drug, A, B, or C, and they don't know if it is going to help or not. They also know that they are starting another round of painful experiences, which during the TV, was the most painful part of the visit. Thus, it is possible that now they are starting to have a little more anxiety, based on that earlier experience. Anticipation and increased uncertainty can raise SCL. Thus, these possibilities could raise baseline SCL for Cold2 during A, B, and C, making it higher than baseline for Cold1 on that visit. We note that the method still works across these variable conditions.

Anchoring the measurements to the three regions defined in this work addresses the specificity problem and using the deltas between these regions provides significant discrimination even with a small number of people (n = 23). Most other measures, such as the absolute ratings given subjectively on an eVAS, require much larger groups of people to achieve significant discriminability. Thus, the new method appears to provide a scientifically significant advantage AND to provide practical cost-saving improvements over the commonly used visual-analog scale. While both are specific to a pain event, the combination used in our method can achieve the level of discrimination desired between study conditions, using a trial with a smaller group of participants.

While the new measure has shown significant performance across many challenging comparisons, this work has limitations. The participants were all healthy adult males, who had to be capable of self-reporting their peak pain moment; thus, this method would not work with infants or others who could not communicate their pain, although adaptations of it may still be useful in such cases (43). It is also unclear how it would work in women; a larger study that examines their physiological changes across months exhibiting the variety of hormonal changes is needed. This work did not have the resources to address the larger study required to control for hormonal influences. The three-region method also requires knowing the timing for the onset of a pain stimulus, which is not likely to be present in all situations, although it can be controlled in many clinical trial studies. While we use a capsaicin injection to model chronic pain, it is also important to examine long-term real-world chronic pain, where we might expect to see significant baseline shifts as well as asymmetries in EDA, and measure also effects on daily behaviors, including pain's impact on sleep and activity patterns and how these relate to the EDA measures (41, 44, 45).

In the future, there are many possible ways to extend and possibly improve upon this work. We decomposed the tonic and phasic EDA data in a very basic, traditional way, and today there is a growing literature describing more advanced ways to analyze components of EDA data, e.g. see the systematic review by Posada-Quintero and Chon (46) especially contributions examining narrower frequency bands that may improve upon our use of a simple low-pass filter to give even better results (47). These additional ways to process the data would be interesting to explore. Many lab-based studies using heat-based pain induction have found that processing the EDA to extract the more rapid SCR's provides more accurate estimation of the pain level than does using the SCL. Particularly intriguing are findings from Posada-Quintero et al. showing that using a narrower frequency band inspired by analyzing sympathetic nervous system activity (similar to in heart-rate variability studies) leads to improved results for estimating the pain stimulation intensity (48, 49). Their findings also showed that EDA was better at estimating the stimulus intensity than the subjects' self-report scores. Our work is not directly comparable because we are not trying to use EDA to estimate self-report level, nor are we trying to estimate the numeric intensity level of a pain stimulus; instead, we are trying to examine, following a typical clinical trials protocol for evaluating a new treatment, if it is showing a significant difference in pain reduction compared to placebo or to no treatment. Our work doesn't directly use self-reported “level of pain” other than as an intermediate step to locate the self-reported moment of peak pain, which is then used to bound a region for measuring the skin conductance.

Despite the different goals of this work, an important future direction is to closely examine the contributing features of an EDA signal, including content from different frequency bands, and how they relate to (1) distress or discomfort, which would be expected to be higher for a first visit (expected in the TV condition and untreated or anticipated-as-untreated conditions), and to (2) characteristics of different pain models and their stimuli. Many studies include repeated stimuli such as electrical or heat pulses, each of which can elicit an anticipatory orienting response in the EDA (these were unable to be examined in our study, because their stimuli were not timed properly for allowing synchronization to EDA). Stress and orienting responses, and small movements they elicit, may confound the pain response. At the same time, several researchers have shown that the high-speed fluctuating changes in EDA have been some of the most valuable features for classifying pain-related distress, (50), levels of self-reported pain, e.g. (51), and objective levels of applied pain intensity (48, 52). The latter have used particularly novel and well-performing frequency-specific methods of extracting SCR's, which would be important to examine in future work. We did not compute SCR's in this work, despite that we initially expected that they would be more informative than SCL, especially with our many-visit study design, since a mean SCL can vary highly within a person across days. Upon inspecting our data, even before smoothing, we observed very few SCR's, and many cases with zero SCR's, even during strongly-reported pain. See, as exemplary, the relatively smooth examples in Figures 2, 3, where there is a dominant change in SCL after the onset of the pain stimulus, but with very few fluctuations around the large rise. In short, counting SCR's in the traditional way would not have given significant pain discrimination in this clinical trial. At the same time, our method does not use SCL in the traditional way, where typically the SCL after the pain stimulus is compared across treatments. That approach does not show a significant difference for analgesics vs. non-treatment or placebo in our study (in part because the opioid increased the SCL). Instead, our method anchors SCL's region of computation specifically to the pain event, normalizes it across a day's session, and computes changes in levels across three regions in a way that apparently reduces its influences from other factors that may have caused “average SCL” to not perform discriminatively in past studies. The new method's results outperform eVAS for discriminating the effects of analgesic vs. placebo under gold-standard blinded test conditions in a professionally-conducted clinical trial.

One might ask “Why compute three regions when only one measure of pain is typically sought?” Indeed, if a medical professional wants to know quickly whether or not a patient is hurting badly, asking for a subjective report is faster and if the patient can provide it, it can suffice for triage. However, a better characterization is needed in clinical trials to examine if one treatment reduces pain more than another. For clinical trials, the proposed new measure provides a better result than using eVAS. The new measure provides objective physiology data, anchored specifically in an onset-of-pain event and a subjectively-timed “peak experience of pain” event, establishing quantitative changes in the anticipatory, peak pain, and recovery regions of the pain experience. All three regions may be targets for future improved treatments. Our study shows that different treatments may affect these regions differently, and that quantifying these three regions in the way described provides greater discrimination of treatment effects than using self-reported pain.

Future work might examine, for different pain models, which way to use the three regions to give the best discrimination. As seen for capsaicin (Figure 7), the biggest differences between the no-treatment or placebo conditions and the two analgesics occur in Region II. If we were to simplify the three-region model to a two-region model (combining Region II and III, and comparing their combined value to that of Region I) then the size of the effect will be reduced, even if in some cases the difference is still significant. For the cold pressor model, it is not Region II but it is Region III that shows the biggest difference; this can be visualized in Figure 4, by shifting the red plots down to match their normalized SCL to that of Cold1 in Region I. The differences are due to the different pain models: capsaicin pain peaks immediately, while cold pressor pain takes minutes before it climbs. By attending to where the regions are most likely to differ for a given pain model, it becomes possible to examine more precisely where the benefits of a treatment occur.

Overall, our work contributes to the important goal of improving the measurement of pain, not trying to make it completely objective or deny its subjective reality, but making it *more* objective, and making its quantification more specific to three regions of a dynamic pain experience. The method is low-cost, practical, and easily combined (if desired) with studies that use more costly measures such as fNIRS, fMRI, and new kinds of brain imaging. The new method is well-suited for studies that evaluate different treatments for pain, such as clinical trials. Not only does the new method provide better discriminability than eVAS with fewer participants, it reduces the psychological and ethical costs of inflicting pain on a larger than necessary group of people. While the results we have shown suggest that the new method is more sensitive than traditional eVAS for clinical trials, our work has not focused on what is the underlying line of action. The evidence of a more sensitive measurement, compared to eVAS, showing the effects of the two analgesic drugs used, especially pregabalin, may be related to its possible action directly on the sympathetic nervous system rather than specifically targeting the perception of pain. More work is needed to understand how effective the proposed measure continues to be when tested with additional kinds of pain models and treatments. The three-region method also may potentially improve the methodology for studies designed to elicit and measure responses to pain by giving better insights into placebo interactions and the impact of cognitive and affective contexts that can influence the experience of pain, whether these occur before the onset of the actual pain stimulus, after its onset, or during the recovery period after the peak pain. Overall, this study, within the format of a clinical trial, has shown that the proposed method works better than eVAS across multi-day visits by healthy men, across two pain models, and across conditions of no-treatment, placebo, and two well-known effective analgesics.

## Data Availability Statement

The study participants were in Europe and while they consented to have their anonymized data published as part of this research, they did not consent to have their raw data shared for other research purposes; thus, under European law, their datasets cannot be made available.

## Ethics Statement

The studies involving human participants were reviewed and approved by ICON plc IRB, Manchester, UK. The patients/participants provided their written informed consent to participate in this study.

## Author Contributions

VB contributed the idea and first implementation of anchoring the pain measurement timing to the peak self-reported pain, conducted data analyses, and helped write the paper. RP contributed to the design of the study, the methods of data analysis, and wrote most of the paper. CS contributed to the design of the study, writing the trial protocol, obtaining approval by ethics committees and medicines agencies in Manchester, helping write the paper, and was part of training the CRO performing the trial and conducting clinical oversight with the trial. All authors contributed to the article and approved the submitted version.

## Funding

This work received funding from Grunenthal GmbH. The study was co-designed by author RP (working at MIT and consulting for Affectiva at the time) and by author CS (working for Grunenthal at the time). The study and all data collection were performed by ICON, independent of Grunenthal and Affectiva. The funder did not influence data collection, analysis or interpretation of the data, or influence the writing of this article or the authors' decision to submit it for publication. CS was no longer affiliated with Grunenthal at the time that she assisted in the preparation of this manuscript for submission.

## Conflict of Interest

RP is employed by MIT as a full professor and consults for Empatica Inc, where she is a co-founder and shareholder, serves as Chairman of the Board and consults part-time as Chief Scientist. RP receives royalties on patents for her AI-related and wearables inventions owned by MIT. RP also serves as an expert witness representing Apple, Inc. in matters related to wearable technologies. At MIT, her lab's research receives funding from the NIH via the Massachusetts General Hospital Depression Research Clinic and from a consortium of over fifty companies listed at https://www.media.mit.edu/posts/member-companies. She receives speaker fees through Stern Strategy. These relationships present no conflict of interest at the time of this paper submission. VB is self-employed. CS is employed by Novo Nordisk A/S. CS was employed by Grünenthal GmbH during the design and conduct of the trial. This study received funding from Grünenthal GmbH. The funder had the following involvement with the study: assisting in designing the trial, overseeing the trial conduct, and engaging in discussions around the data analysis.

## Publisher's Note

All claims expressed in this article are solely those of the authors and do not necessarily represent those of their affiliated organizations, or those of the publisher, the editors and the reviewers. Any product that may be evaluated in this article, or claim that may be made by its manufacturer, is not guaranteed or endorsed by the publisher.
